# The physical activity paradox revisited: a prospective study on compositional accelerometer data and long-term sickness absence

**DOI:** 10.1186/s12966-020-00988-7

**Published:** 2020-07-20

**Authors:** Nidhi Gupta, Sofie Dencker-Larsen, Charlotte Lund Rasmussen, Duncan McGregor, Charlotte Diana Nørregaard Rasmussen, Sannie Vester Thorsen, Marie Birk Jørgensen, Sebastien Chastin, Andreas Holtermann

**Affiliations:** 1 National Research Centre for the Working Environment, Lersø Parkalle 105, DK-2100 Copenhagen Ø, Denmark; 2 Business Information & Analytics, Copenhagen Business School, Solbjerg Plads 3, DK-2000 Frederiksberg, Denmark; 3 Department of Public Health, Section of Social Medicine, University of Copenhagen, Copenhagen, Denmark; 4 School of Health and Life Science, Glasgow Caledonian University, Cowcaddens Road, Glasgow, G4 0BA Scotland; 5 Biomathematics and Statistics Scotland, JCMB, The King’s Buildings, Peter Guthrie Tait Road, Edinburgh, EH9 3FD Scotland, UK; 6 Occupational Health and Safety, Department of Ergonomic and Technical Counselling, Municipality of Copenhagen, Copenhagen, Denmark; 7 Department of Movement and Sport Sciences, Ghent University, Ghent, Belgium; 8 Department of Sports Science and Clinical Biomechanics, University of Southern Denmark, Odense, Denmark

**Keywords:** Physical activity, Sedentary behavior, Accelerometers, Sick leave, Occupational health, Time-use epidemiology, Register-based sickness absence

## Abstract

**Background:**

The ‘physical activity paradox’ advocates that leisure physical activity (PA) promotes health while high occupational PA impairs health. However, this paradox can be explained by methodological limitations of the previous studies—self-reported PA measures, insufficient adjustment for socioeconomic confounding or not addressing the compositional nature of PA. Therefore, this study investigated if we still observe the PA paradox in relation to long-term sick absence (LTSA) after adjusting for the abovementioned limitations.

**Methods:**

Time spent on moderate-to-vigorous physical activity (MVPA) and remaining physical behaviors (sedentary behavior, standing, light PA and time in bed) at work and in leisure was measured for 929 workers using thigh accelerometry and expressed as isometric log-ratios (*ilrs*). LTSA was register-based first event of ≥6 consecutive weeks of sickness absence during 4-year follow-up. The association between *ilrs* and LTSA was analyzed using a Cox proportional hazards model adjusted for remaining physical behaviors and potential confounders, then separately adjusting for and stratifying by education and type of work.

**Results:**

During the follow-up, 21% of the workers experienced LTSA. In leisure, more relative MVPA time was negatively associated with LTSA (20% lower risk with 20 min more MVPA, *p* = 0.02). At work, more relative MVPA time was positively associated with LTSA (15% higher risk with 20 min more MVPA, *p* = 0.02). Results remained unchanged when further adjusted for or stratified by education and type of work.

**Conclusion:**

These findings provide further support to the ‘PA paradox’.

## Background

Physical activity (PA) reduces the risk of chronic diseases and mortality [1]. However, research indicating the health benefits of PA is predominantly limited to the leisure domain- a time period in a day where PA occurs during domestic work, transport or spare time [2]. Adults engage in PA at work⁠—a domain where individuals spend a half of their awake time. However, there is no consistent documentation of a beneficial health effect of occupational PA (OPA) [3–6]. In fact, a recent meta-analysis of almost 200,000 participants observed an increased risk of all-cause mortality among males with high OPA [7]. These potential contrasting health effects of PA at work and in leisure domains—that is PA at work is detrimental while PA in leisure is beneficial for health—is termed ‘the physical activity paradox’. The PA paradox has recently received extensive attention in the field of PA and health [8, 9].

In particular, researchers have suggested that the PA paradox is merely a result of methodological limitations of existing studies [9]. One such limitation lies in the measurements of physical behaviors, like the use of self-reported information on physical behaviors that has been found to be imprecise and potentially biased [10, 11]. Besides this, existing prospective studies on the PA paradox have disregarded the compositional nature of time-use data like physical behaviors [12–15]. The compositional nature of physical behaviors data means that the longer time spent on a specific physical behavior, such as moderate-to-vigorous PA (MVPA), will consequentially leave less time spent on other physical behaviors, such as light PA (LIPA), sedentary behavior or sleep. To counter this challenge, the time-use data on physical behaviors should be analyzed using a Compositional Data Analysis (CoDA) approach [12–14, 16]. Another limitation is the potentially inadequate adjustments for socioeconomic status (SES) confounding, where analyses of homogeneous groups with respect to socioeconomic characteristics are preferable [9].

The PA paradox has been shown to be associated with long-term sickness absence (LTSA) — an established predictor of all-cause mortality [17], chronic disease [18], and early exit from the labor market [19–22] with considerable economic burdens on companies and society [23, 24]. Studies have shown that high levels of OPA increase risk of prospective LTSA [3, 25] while high levels of leisure time PA decrease this risk [3]. However, the present study addresses, for the first time, the previous limitations of these studies by using device-based measures of physical behaviors at work and in leisure, addressing the compositional nature of physical behaviors data, and adjusting for SES confounding. Thus, the aim of this study was to investigate if we still observe the PA paradox related to LTSA after addressing the abovementioned limitations of related previous studies. We hypothesized that higher relative time spent on MVPA at work will increase the risk of LTSA while higher relative time spent on MVPA in leisure will decrease this risk among workers.

## Methods

### Data and study population

The present study is based on the prospective data from the ‘technically measured compositional Physical wOrk DEmands and Prospective register-based Sickness Absence study (PODESA) cohort [15]. This cohort was formed by harmonizing data from two cohorts, the ‘Danish Physical ACTivity cohort with Objective measurements’ (DPhacto) [26] and the ‘New method for Objective Measurements of physical Activity in Daily living’ (NOMAD) cohort [27]. Recruitment of the workplaces was performed in collaboration with the labor unions. Labor unions chose 22 workplaces that were offered participation. NOMAD cohorts included workers from seven workplaces primarily engaged in construction, cleaning, garbage collection, manufacturing, assembling, mobile plant operation and in the health service sector. DPhacto cohort included workers from 15 workplaces engaged in cleaning, manufacturing and transport sector. Previous studies have shown no clear difference between participants and non-participants in the NOMAD and DPhacto cohorts [26, 28].

The baseline data in NOMAD and DPhacto cohorts were collected between 2011 to 2012 and 2012 to 2013, respectively. Both cohorts used similar procedures of 24-h time accelerometry and comprised mainly blue-collar workers in Denmark, enabling the harmonization. More details on the setting, locations, recruitment, and inclusion and exclusion criteria in these cohorts and on the harmonizing procedures can be found elsewhere [15].

The data on LTSA during 4 year follow-up from the date of completing the baseline was retrieved from the Danish national Register-based Evaluation of Marginalization (DREAM [19]).

Representatives for the participants, that is, the management and worker unions, were actively involved in the planning, design, decision on measurements, recruitment of the workplaces, data collection, feedback to participants, and interpretation and dissemination of the results.

### Accelerometry at work and in leisure

Workers wore a thigh-based triaxial ActiGraph GT3X+ accelerometer (Florida, U.S.A) for 24 h for up to five workdays [27, 29]. Simultaneously, during those five days, workers also filled-in a diary reporting their time of starting and ending work and going to and out of the bed each day, time of reference measurement, and non-wear periods. The accelerometry data were downloaded using ActiLife Software version 5.5 [30] and further processed using a MATLAB program Acti4 [31, 32]. Acti4 has previously shown a high sensitivity and specificity in detecting PA at work and in leisure [31]. Acti4 was used to determine time spent sedentary (sitting and/or lying), standing still, moving (standing with slight movements), walking slow (< 100 steps per min) and fast (≥100 steps per min), running, cycling and stair climbing [31]. For the analysis, time spent moving and slow walking was merged to calculate light physical activity (LIPA), while time spent on fast walking, stair climbing and running was merged to calculate moderate-to-vigorous physical activity (MVPA) [33]. Leisure MVPA also included cycling time [33]. Diary-based information was used to determine time in bed⁠—a period between going to and out of the bed that were further visually checked for verification in the Acti4. A work period was defined as self-reported working hours spent on primary occupation while leisure period was defined as non-work periods (including domestic work and transport), excluding time in bed.

All non-work days and accelerometry non-wear periods were excluded. The criteria to identify the non-wear periods were as follows: (a) the Acti4 detected periods longer than 60 min showing zero counts per minute, (b) workers reported non-wear periods in the diary and (c) detection of artefacts or missing data via visual inspection of the accelerometer data.

Workers who had at least one day with valid work, leisure, and time in bed periods were involved in further analyses. A work or a leisure period was considered valid if it comprised ≥4 h of wear time or ≥ 75% of the average wear-time across days, respectively [16, 26, 34, 35]. A time in bed period was considered valid if it comprised at least 4 hours of measurements [34]. 

The mean time spent sedentary, standing and on LIPA, MVPA and median time spent in bed on all valid days were calculated to express average daily work and leisure physical behaviors [33, 34].

### Prospective register-based long-term sickness absence

Four-year prospective data on LTSA was retrieved from the DREAM register [36]. This register contains weekly information on granted subsidized sickness absence for each individual in Denmark. The sickness absence compensation is given to the employer who can claim a refund from the state after 30 days of sickness absence. Therefore, DREAM contains information on sickness absence periods of ≥5 consecutive weeks. LTSA was defined as the occurrence of the first (if any) ≥6 consecutive weeks of sickness absence period during the 4-year follow-up from the date of completing the baseline measurements. We selected this cut off point based on previous research [37]. The data on sickness absence benefit from the DREAM register have shown excellent accuracy when compared to companies own records of employees' sickness absence [38].

### Potential confounders

We chose confounders a priori based on studies on the association between occupational and leisure time physical behaviors and sickness absence [3, 39, 40]. Potential confounders were age, sex, body mass index (BMI), smoking status, duration of occupational lifting and carrying, and education and type of work as proxy indicators of SES. Age was determined using workers’ unique civil registration number. Sex of the workers was determined using single item “are you male or female?”. Workers’ height and weight were objectively measured by the trained personnel to determine their BMI (kg/m^2^). Smoking status was determined using a single item with response categories summarized to smokers (smoking daily or sometimes) and non-smokers (ex-smokers and never smoked). Occupational lifting and carrying duration was determined using a single item with 6 responses ranging from ‘almost all the time’ to ‘never’ [33]. The information on workers’ education and type of work was included as indicators of SES [41, 42]. The education of the workers was determined using a single item “are you skilled or unskilled?”. The information on type of work was collected using single item “are you a worker engaged in administrative work tasks (white collar) or in production (blue-collar)?”. Later, the information on these two measures was summarized in three categories - white-collar, blue-collar skilled, and blue-collar unskilled.

### Statistical analyses

The statistical analyses were performed using R software (version 3.5.1, R Foundation for Statistical Computing, Vienna, Austria) using the software package ‘robcompositions’ [43] and ‘survival’ [44].

The data were analyzed according to the CoDA approach [45]. First, the four-part time composition of work (MVPA, sedentary, standing, and LIPA) and five-part time composition of leisure (MVPA, sedentary, standing, LIPA, and time in bed) were expressed as isometric log-ratios (*ilrs*). The first *ilr* coordinate for the work and leisure composition represents time spent on MVPA relative to the geometric mean of remaining behaviors. In subsequent *ilrs*, the denominator of the first *ilr* was further split to create remaining *ilrs* [46]. We created *ilrs* by treating work and leisure time as two separate compositions instead of considering them as two sub compositions of a whole day. The reason for this is that (a) generally the time spent at work and in leisure are fixed and (b) physical behaviors at work and in leisure are a result of different purpose, context and environments. Therefore, it is rarely possible to allocate time spent on physical behavior between domains. However, statistically, our results were similar irrespective of how we treat the time domains to create *ilrs* (see the results in the Additional file 1).

The Cox proportional hazards regression model was used to analyze the association between *ilrs* (i.e. the log-transformed work and leisure compositions) as explanatory variables and the onset of LTSA as the dependent variable. Hazard ratios (HRs) were estimated by maximizing the partial likelihood function [47].

In the cox regression, workers contributed with the risk time till the event of LTSA has occurred or till the end of the follow up (4 years) if the event has not occurred. Some workers could not be followed-up for the entire 4-year follow-up time in the DREAM register due to following reasons: emigrated, died, entered early retirement, entered ordinary retirement, or became pregnant (measured as going on maternity leave 8 months later and being a woman). These workers were ‘censored’ in the analyses at the time when one of these reasons occurred, and contributed with “time at risk” only up to this point in time.

The Cox regression model was adjusted for age, sex, BMI, smoking status, occupational lifting/carrying duration, and MVPA and other physical behaviors in the mutual domain (sets of *ilrs* at work and in leisure were entered together in the model). The assumptions of proportional hazards were met when tested by visual inspection and using the Grambsch-Therneau test [48]. The model coefficient for the *ilrs* were assessed using Wald test statistics (*z*) and the associated probability of type I error (*p*), considering *p* < 0.05 to indicate a statistically significant relationship.

Of the total 929 workers, 118 workers had missing SES data (in three categories: white-collar, blue-collar-skilled, and blue-collar-unskilled). On the remaining 811 workers, we performed following two analyses to test if the main results were independent of SES confounding; (1) adjusting for SES: we performed the analyses without and with additional adjustment for SES and (2) stratification of the analyses on the three categories of SES. The reason behind performing both adjustment and stratification on SES in the analyses was to thoroughly understand if SES confounds the intended association of interest.

#### Effect size interpretation

To interpret the strength of the association, procedures explained in previous studies were used [33]. First, sample compositional mean of all physical behaviors at work and in leisure was calculated (Table 1). Based on the compositional means, new work and leisure time compositions of MVPA and other behaviors were created by incrementally increasing/decreasing the time spent on MVPA and other behaviors while keeping the total time at work and leisure constant. Thereafter, the Cox parameter estimates were used to predict the difference between risk of LTSA, expressed as a hazard ratio (HR), associated with the new work and leisure time compositions and the sample compositional mean. Finally, the predicted HRs against reallocations (in minutes) at work and leisure were plotted. The corresponding 95% CI of the predicted HR are presented in Additional file 2.

**Table 1:** Descriptive statistics for 929 workers involved in the analyses

Variables	n	Mean (SD)	%
Age (years)	929	44.9 (9.7)	
Females	418		45
BMI (kg/m^2^)	929	27.1 (4.8)	
Non-smokers	646		70
White-collar	154		17
Occupational lifting/carrying duration (1–6)^a^	929	3.9 (1.5)	
SES			
White-collar	154		19
Blue-collar skilled	320		39
Blue-collar unskilled	337		42
Compositional means of time spent on physical behaviors (mins)			
Work			
MVPA	929	64	
Sedentary	929	176	
Standing	929	137	
LIPA	929	74	
Leisure			
MVPA	929	33	
Sedentary	929	311	
Standing	929	77	
LIPA	929	41	
Time in bed	929	429	

^a^1 = almost all the time, 6 = never; the time composition of physical behaviors at work and in leisure do not add up to 1440 min (24 h) due to non-wear time of ~ 1.6 h

As we calculated the *ilrs* by treating physical behaviors at work and in leisure as two separate compositions, we closed the geometric means to the average work and leisure time instead of closing to 1440 min

*Abbreviations*: *BMI* Body mass index, *LIPA* Light physical activity, *LTSA* Long-term sickness absence, *MVPA* moderate-to-vigorous physical activity *SES* socioeconomic status

## Results

**Fig. 1:**
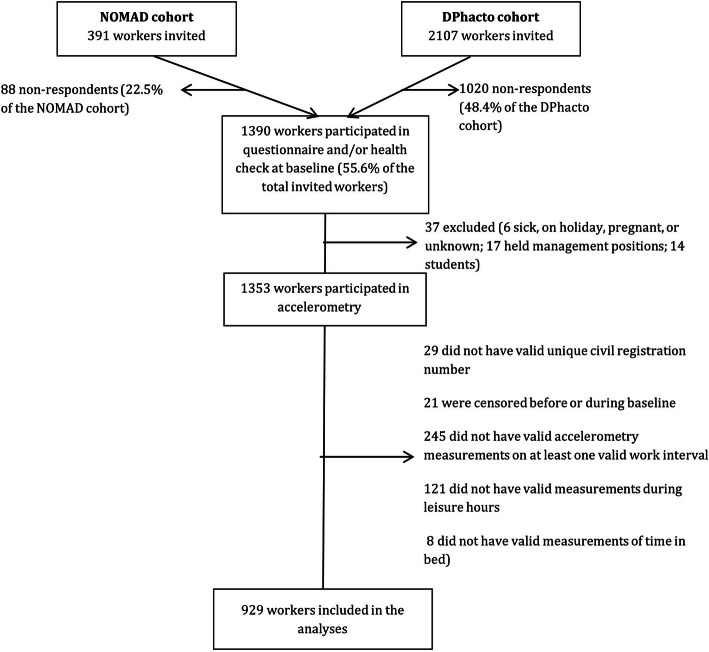
Flow of the participants in the study

Out of the 2498 eligible participants, 929 (37%) workers had sufficient data to be involved in the analyses. A detailed flow chart is shown in Fig. 1.

Table 1 shows the descriptive statistics of the 929 workers involved in the analysis. The participants were on average 45 years old with a BMI of 27 kg/m^2^. Fifty-five percent of them were men, and 30% of them smoked.

Of the 929 workers included in the analyses, 191 (21%) had an event of LTSA in the 4-year follow-up (that is 212 weeks). The median time to an LTSA event was 89 [interquartile range (IQR) =98.5] weeks. Of the remaining who did not have an event of LTSA, forty-seven (5%) workers were censored (see reasons for the censoring in the methods section) over the 4-year follow-up period with an average follow-up time of 94 (IQR = 101) weeks.

Workers wore accelerometers for, on average, 1343 min (SD = 104 min) per day that was divided into work (M = 451 min, SD = 80 min) and leisure (M = 892 min, SD = 109 min). The minimum wear time during work and leisure domain were 223 min and 536 min, respectively.

**Fig. 2:**
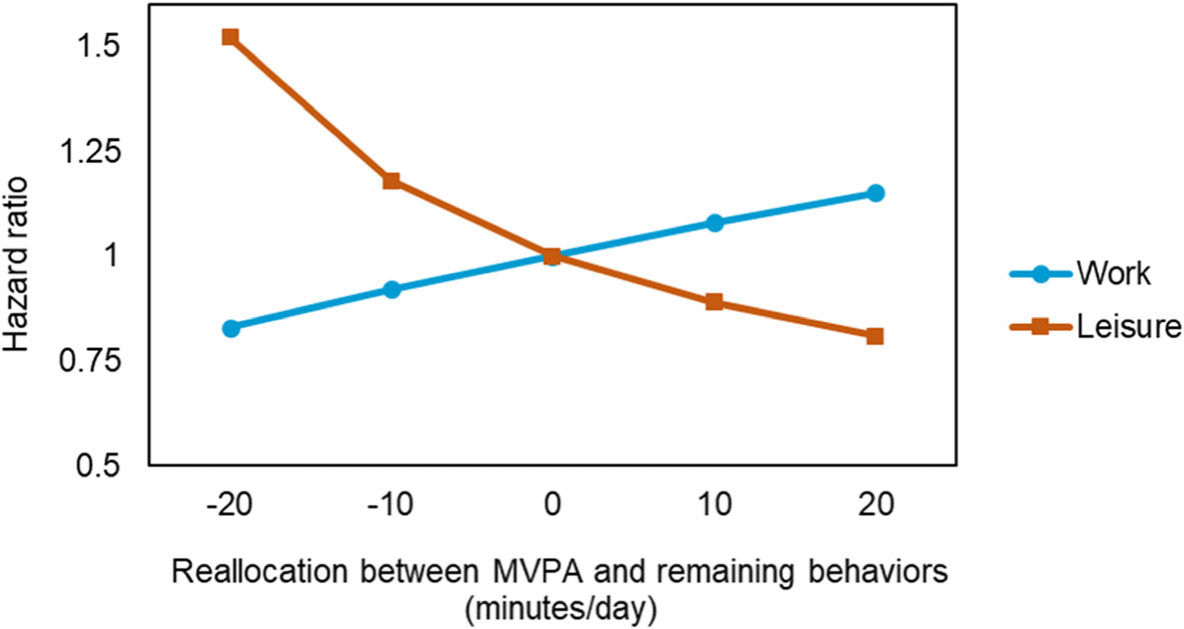
Reallocations of time between MVPA and remaining behaviors at work and in leisure and their association with the risk of long-term sickness absence: Results of a compositional isotemporal substitution analysis based on the Cox proportional hazards model. ‘0’ on the x axis represents the average composition (work: 64 min MVPA, 176 min sedentary, 137 standing and 74 min LIPA; leisure: 33 min MVPA, 311 min sedentary, 77 min standing, 41 min LIPA, and 429 min time in bed). The hazard ratio indicated the difference between the risk of LTSA associated with new reallocated compositions and average compositions

The results of the Cox proportional hazards models are shown in Additional file 3. Specifically, more time spent on MVPA at work, relative to other work behaviors, was significantly positively associated (*p* = 0.02) while more time spent on MVPA in leisure, relative to other leisure behaviors, was significantly negatively associated (*p* = 0.02) with LTSA. Figure 2 shows that, for example, reallocating 20 min to MVPA at work from the remaining work behaviors was associated with ~ 15% higher risk of LTSA while reallocating 20 min to MVPA in leisure from remaining leisure behaviors was associated with ~ 20% lower risk of LTSA.

Results of the sensitivity analysis are shown in Additional file 4. Adjusting for SES did not change the main results of the association between relative MVPA in both domains and LTSA (without adjusting for SES; work, *z* = 2.68, *p* = 0.01, leisure, *z* = − 2.02, *p* = 0.04, with adjustment for SES; work, *z* = 2.73, *p* = 0.01, leisure, *z* = − 2.04, *p* = 0.04). Additionally, the direction of the estimates was similar to the primary analysis when stratifying by the three categories of SES.

## Discussion

Our study showed that relative time spent on MVPA in leisure reduces the risk of LTSA, while relative time spent on MVPA during work increases the risk. These results support the existence of the ‘PA paradox’.

In leisure, more time spent on MVPA relative to other physical behaviors (sedentary, stand, LIPA and time in bed) was significantly associated with lower risk of LTSA. For example, reallocating 20 mins to MVPA from other leisure behaviors was associated with 20% lower risk of LTSA. This observation of a beneficial association of PA in leisure with LTSA is in accordance with the results of existing studies using self-reported measures of PA [49] and not applying the CoDA approach [7]. The potential mechanisms behind benefits of leisure time PA could be through improved health and physical capacity [50, 51], making the workers better perform their work tasks. Overall, we observed that reallocating just a little duration, for example 5 min, to MVPA from other behaviors seem to lower the risk of LTSA. Increasing a little duration of MVPA (defined as time spent fast walking, stair climbing, running, and cycling) could be feasible for many workers and can be facilitated by modifying the structural environment (eg., more bike lanes) or work environment [eg., work tasks offering restitution, likely giving energy and motivation to workers to perform leisure MVPA [52]]. A slightly lowered risk for LTSA can have enormous effects on reducing economic costs for companies and the society, as well as for the individual, since LTSA often leads to unemployment and further aggravation of health and life-situation crisis [53, 54].

At work, more time spent on MVPA relative to other physical behaviors was positively associated with LTSA. For example, reallocating 20 mins to work MVPA from other work behaviors was associated with 15% higher LTSA risk. No previous studies on the association between work physical behaviors and LTSA have used device-measured physical behaviors, like accelerometers, and a CoDA approach with prospective register-based LTSA information. Thus, we cannot directly compare the estimates of our study with previous studies [3, 7]. Nevertheless, the overall finding of an increased risk for future LTSA with higher levels of work MVPA is in line with some studies based on self-reports [3, 7]. The potential mechanism behind our finding could be that work MVPA is influenced by different constraints and has different characteristics than leisure MVPA [55]. Work MVPA is performed mainly to complete working tasks and compared with leisure, there is a limited possibility of tailoring the duration, intensity, and variation of the MVPA according to the individual needs and preferences. Because of these constraints, the work MVPA can lead to excessive exertion and fatigue without sufficient time for recovery [56], which over time can increase risk of impaired health and LTSA [57, 58].

We also observed that our results did not substantially change when the analyses were adjusted for SES indicators. Studies testing the PA paradox have been criticized for not adjusting for the SES confounding [9]. To address this limitation, we performed the analyses without and with adjustment for a proxy measure of SES (three categories: white-collar, blue-collar-skilled, and blue-collar-unskilled) and even stratified the analyses on these categories. We still observed the PA paradox even after these adjustments and stratifications based on SES, confirming that PA paradox exists independent of SES of workers.

### Strengths and limitations

The main strengths of the study are the thigh-worn accelerometry-based physical behaviors data that have shown to have high reliability and adequate validity [31, 59]. Another strength is the use of CoDA which adequately handles the compositional structure of time-use data of physical behaviors [12, 45]. Additionally, this study adjusted for remaining physical behaviors (sedentary behaviors, standing and LIPA and time in bed) within 24 h. Another strength was the use of national register data with valid prospective measures of LTSA [36]. Finally, the opportunity to adjust for possible SES confounding when testing the PA paradox was another strength of the study.

We used proxy measure of education and type of work indicating workers’ SES. Therefore, a better measure of SES confounding such as data from national registers on household income, job group, and education (Statistics Denmark [https://dst.dk/da]) are needed in the future to confirm these findings. Similar future studies should also focus on testing the PA paradox in relation to the other outcomes, such as mortality. Another limitation of this study was the lack of objective information on other occupational physical behaviors such as lifting and on the context in which the physical behaviors occur. Additionally, we also lacked information on lunch breaks at work. In many countries and between industries within countries, it varies if the lunch break is paid or not. Thus, information on lunch time might have helped to better separate physical behaviors occurring during work and leisure time.

### Practical recommendations

LTSA is an early antecedent of impaired health with an extensive economic burden on workplaces and society [23, 24]. Moreover, LTSA can have enormous consequences for the individual workers, as LTSA is a strong predictor of premature exit from the labor market [19, 21] and mortality [60]. Given that PA at work and in leisure are modifiable factors, the findings of the present study can be of importance for better prevention of LTSA with systemic interventions in both work and leisure environments. These interventions should be accompanied by appropriate environmental and structural changes at work and in leisure, ensuring success in modifying physical behaviors of the workers.

## Conclusion

In conclusion, our study suggests that MVPA in leisure reduces the risk of LTSA, while MVPA during work increases the risk of LTSA. This finding supports the PA paradox.

## Supplementary information

**Additional file 1:** Comparison of the results of the study using two different kind of *ilrs*; (a) work and leisure domain treated as two separate compositions and (b) work and leisure domains treated as two sub compositions of a whole day composition. **Additional file 2:** The 95% confidence intervals of the difference in the predicted hazards corresponding to the new work and leisure time compositions and the sample compositional mean at work and leisure. **Additional file 3:** Results of Cox Proportional Hazard model indicating the association between composition of relative MVPA at work and leisure and risk of long-term sickness absence among 929 workers. **Additional file 4:** Results of the sensitivity analyses adjusting for indicators of SES (type of work and education) and stratifying on three categories of SES on 811 workers who had data SES.

## Data Availability

The fully anonymized data from the baseline in NOMAD and DPHACTO from each participant involved in the main analysis of this study are available in a Danish public repository DPhacto: (http://dda.dk/catalogue/28618?lang=en, NOMAD: http://dda.dk/catalogue/28617?lang=en). The fully anonymized data on prospective long-term sickness absence is available upon request from statistics Denmark (A Central Authority on Danish Statistics: https://dst.dk/da).

## Abbreviations

BMI: Body mass index
CoDA: Compositional data analysis
DPhacto: Danish Physical ACTivity cohort with Objective measurements
HR: Hazard ratio
LIPA: Light physical activity
LTSA: Long-term sickness absence
MVPA: Moderate-to-vigorous physical activity
NOMAD: New method for Objective Measurements of physical Activity in Daily living
OPA: Occupational physical activity
PA: Physical activity
PODESA: Physical wOrk DEmands and Prospective register-based Sickness Absence study
SES: Socioeconomic status
