# Supplementary material for: The physical activity paradox revisited: a prospective study on compositional accelerometer data and long-term sickness absence
Source: Int J Behav Nutr Phys Act. 2020 Jul 20;17:93. doi: 10.1186/s12966-020-00988-7 (PMC7370435; doi:10.1186/s12966-020-00988-7)
Supplement: Supplementary file 1 — Additional file 1: Comparison of the results of the study using two different kind of ilrs; (a) work and leisure domain treated as two separate compositions and (b) work and leisure domains treated as two sub compositions of a whole day composition. Additional file 2: The 95% confidence intervals of the difference in the predicted hazards corresponding to the new work and leisure time compositions and the sample compositional mean at work and leisure. Additional file 3: Results of Cox Proportional Hazard model indicating the association between composition of relative MVPA at work and leisure and risk of long-term sickness absence among 929 workers. Additional file 4: Results of the sensitivity analyses adjusting for indicators of SES (type of work and education) and stratifying on three categories of SES on 811 workers who had data SES. [file 12966_2020_988_MOESM1_ESM.docx]

Supplementary material

Additional file 1. Comparison of the results of the study using two different set of *ilrs*; (a) work and leisure domain treated as two separate compositions and (b) work and leisure domains treated as two sub compositions of a whole day composition.

We used following balances to create these *ilrs*:

1. Work and leisure as two separate compositions

| *ilrs*/behaviors | MVPA | Stand | LIPA | Time in bed |
| --- | --- | --- | --- | --- |
| Work |  |  |  |  |
| *ilr1* | + | - | - |  |
| *ilr2* | 0 | + | - |  |
| Leisure |  |  |  |  |
| *ilr1* | + | - | - | - |
| *ilr2* | 0 | + | - | - |
| *ilr3* | 0 | 0 | + | - |

+ the behavior is in the numerator of the log ratio, -the behavior is in the denominator of the log ratio, and 0 the behavior is not included in the ratio

1. Work and leisure as two sub compositions of a whole day composition

| ilrs/behaviors | MVPA work | Stand work | LIPA work | MVPA leisure | Stand Leisure | LIPA Leisure | Time in bed |
| --- | --- | --- | --- | --- | --- | --- | --- |
| *ilr1* | + | + | + | - | - | - | - |
| *ilr2* | 0 | 0 | 0 | + | - | - | - |
| *ilr3* | 0 | 0 | 0 | 0 | + | - | - |
| *ilr4* | 0 | 0 | 0 | 0 | 0 | + | - |
| *ilr5* | + | - | - | 0 | 0 | 0 | 0 |
| *ilr6* | 0 | + | - | 0 | 0 | 0 | 0 |

+ the behavior is in the numerator of the log ratio, -the behavior is in the denominator of the log ratio, and 0 the behavior is not included in the ratio

Thereafter, we performed the Cox proportional hazard regression separately for each set of *ilrs* to analyze if the association between physical behaviors at work and leisure and risk of LTSA is dependent on how we create the ilrs. We observed similar direction and strength of the association based on both sets of *ilrs* (see in the table below) - that is more relative MVPA time at work was positively associated with risk of LTSA while more relative MVPA time in leisure was negatively associated with this risk.

| *ilrs* | Work and leisure time as two separate composition | Work and leisure time as two sub compositions |
| --- | --- | --- |
|  | HR (*p*) | HR (*p*) |
| *ilr* (MVPA at work/remaining behaviors at work) | 1.64 (0.02) | 1.64 (0.02) |
| *ilr* MVPA in leisure/remaining behaviors at leisure | 0.62(0.03) | 0.63 (0.03) |

Additional file 2. The 95% confidence intervals of the difference between the risk (expressed as a hazard ratio) of LTSA associated with new reallocated compositions and average compositions.

| Difference in MVPA (min/day) | HR | 95% Lower CI of HR | 95% Upper CI of HR |
| --- | --- | --- | --- |
| Work | | | |
| -20 | 0.83 | 0.71 | 0.98 |
| -10 | 0.92 | 0.86 | 0.99 |
| 0 | 1.00 | 1.00 | 1.00 |
| +10 | 1.08 | 1.01 | 1.15 |
| +20 | 1.15 | 1.02 | 1.30 |
| Leisure | | | |
| -20 | 1.53 | 1.06 | 2.22 |
| -10 | 1.18 | 1.02 | 1.37 |
| 0 | 1.00 | 1.00 | 1.00 |
| +10 | 0.88 | 0.79 | 0.98 |
| +20 | 0.80 | 0.66 | 0.97 |

Additional file 3. The hazard ratios corresponding to each *ilr* obtained from the Cox proportional hazard model among 929 workers.

| Variables | HR | 95%CI Lower | 95%CI Higher |
| --- | --- | --- | --- |
| Work | | | |
| Ilr_1_ | 1.64 | 1.07 | 2.51 |
| Ilr_2_ | 1.12 | 0.94 | 1.34 |
| Ilr_3_ | 1.09 | 0.72 | 1.67 |
| Leisure | | | |
| Ilr_1_ | 0.62 | 0.41 | 0.94 |
| Ilr_2_ | 1.09 | 0.65 | 1.82 |
| Ilr_3_ | 1.28 | 0.69 | 2.38 |
| Ilr_4_ | 1.00 | 0.57 | 1.75 |

*Ilr_1_* = ratio of the MVPA and geometric mean of the remaining behaviors. Ilr_2-4_ are the ratios where the remaining behaviors are further split up; Models adjusted for age, sex, BMI, smoking status, and occupational lifting/carrying duration and MVPA and other physical behaviours in the mutual domain; HR=hazard ratios.

Additional file 4. Results of the sensitivity analyses adjusting for indicators of SES (type of work and education) and stratifying on three categories of SES among workers who had data SES (N=811)

| Analyses | Z | *p* | Z | *p* |
| --- | --- | --- | --- | --- |
|  | Work |  | Leisure |  |
| **Adjustment for SES** |  |  |  |  |
| Without adjusting for SES | 2.68 | 0.01 | -2.02 | 0.04 |
| With adjustment for SES | 2.73 | 0.01 | -2.05 | 0.04 |
| **Stratification based on SES** |  |  |  |  |
| White-collar workers | 1.00 | 0.32 | -1.92 | 0.06 |
| Blue-skilled workers | 1.26 | 0.21 | -1.73 | 0.08 |
| Blue-unskilled workers | 2.24 | 0.02 | -0.64 | 0.52 |

Adjusted for age, sex, BMI, smoking status, and occupational lifting/carrying duration and MVPA and other physical behaviours in the mutual domain.
